# Sugar alcohol degradation in Archaea: uptake and degradation of mannitol and sorbitol in *Haloarcula hispanica*

**DOI:** 10.1007/s00792-024-01365-z

**Published:** 2024-10-28

**Authors:** Marius Ortjohann, Peter Schönheit

**Affiliations:** Institut Für Allgemeine Mikrobiologie, Christian-Albrechts-Universität Kiel, Am Botanischen Garten 1-9, 24118 Kiel, Germany

**Keywords:** Archaea, Mannitol degradation, Sorbitol degradation, *Haloarcula hispanica*, CUT1 ABC transporter, Ketohexokinase from Haloarchaea

## Abstract

The halophilic archaeon *Haloarcula hispanica* utilizes the sugar alcohols mannitol and sorbitol as carbon and energy sources. Genes, enzymes, and transcriptional regulators involved in uptake and degradation of these sugar alcohols were identified by growth experiments with deletion mutants and enzyme characterization. It is shown that both mannitol and sorbitol are taken up via a single ABC transporter of the CUT1 transporter family. Then, mannitol and sorbitol are oxidized to fructose by two distinct dehydrogenases. Fructose is further phosphorylated to fructose-1-phosphate by a haloarchaeal ketohexokinase, providing the first evidence for a physiological function of ketohexokinase in prokaryotes. Finally, fructose-1-phosphate is phosphorylated via fructose-1-phosphate kinase to fructose-1,6-bisphosphate, which is cleaved to triosephosphates by a Class I fructose-1,6-bisphosphate aldolase. Two distinct transcriptional regulators, acting as activators, have been identified: an IclR-like regulator involved in activating genes for sugar alcohol uptake and oxidation to fructose, and a GfcR-like regulator that likely activates genes involved in the degradation of fructose to pyruvate. This is the first comprehensive analysis of a sugar alcohol degradation pathway in Archaea.

## Introduction

Sugar degradation pathways in Bacteria and Eukarya are well studied; e.g. glucose is degraded via the classical Embden-Meyerhof (EM) or the Entner-Doudoroff (ED) pathway. Analyses of sugar degradation in Archaea, the third domain of life, revealed modified versions of the classical pathways (Siebers and Schönheit 2005; Bräsen et al. 2014). In hyperthermophilic Archaea modified EM pathways are predominant, whereas thermoacidophilic Archaea utilize modified ED pathways (Reher et al. 2010; Zaitsev et al. 2018).

In recent years, sugar degradation pathways have been studied in detail in halophilic Archaea, in particular in the model organism *Haloferax volcanii* that utilizes many sugars as carbon and energy source, including various hexoses, pentoses, and deoxysugars. The transport mechanisms of these sugars, their degradation pathways and transcriptional regulation have been elucidated (Pickl et al. 2012; Johnsen et al. 2015, 2023; Sutter et al. 2016; Reinhardt et al. 2019; Tästensen et al. 2020; Kuprat et al. 2021). *H. volcanii* degrades glucose via a semi-phosphorylative ED pathway and fructose via a modified EM pathway. Degradation of fructose in *H. volcanii* involves uptake by a phosphoenolpyruvate-dependent phosphotransferase system (PTS) to form fructose-1-phosphate (F1P), which is the first report of a PTS in Archaea. F1P is further converted via fructose-1,6-bisphosphate (FBP) to triosephosphates involving F1P kinase (1-PFK) and fructose-1,6-bisphosphate aldolase (FBA) (Pickl et al. 2012). Recently, GfcR as a novel transcriptional activator of both glucose and fructose degradation pathways has been identified in *H. volcanii* (Johnsen et al. 2023).

While various sugar degradation pathways have been analyzed in Archaea, little is known about growth on and degradation of sugar alcohols. Growth on the sugar alcohol mannitol has first been reported for *Haloarcula vallismortis* and a degradation pathway has been postulated that involves mannitol oxidation to fructose by mannitol dehydrogenase. Fructose is then phosphorylated by ketohexokinase to F1P, which is further converted to triosephosphates via FBP, involving activities of 1-PFK and FBA (Altekar and Rangaswamy 1992). These enzymes were only partially characterized and encoding genes have not been identified (Krishnan and Altekar 1991; Rangaswamy and Altekar 1994a, b). So far, growth on and degradation of sugar alcohols in hyperthermophilic and thermoacidophilic Archaea have not been reported.

**Fig. 1:**
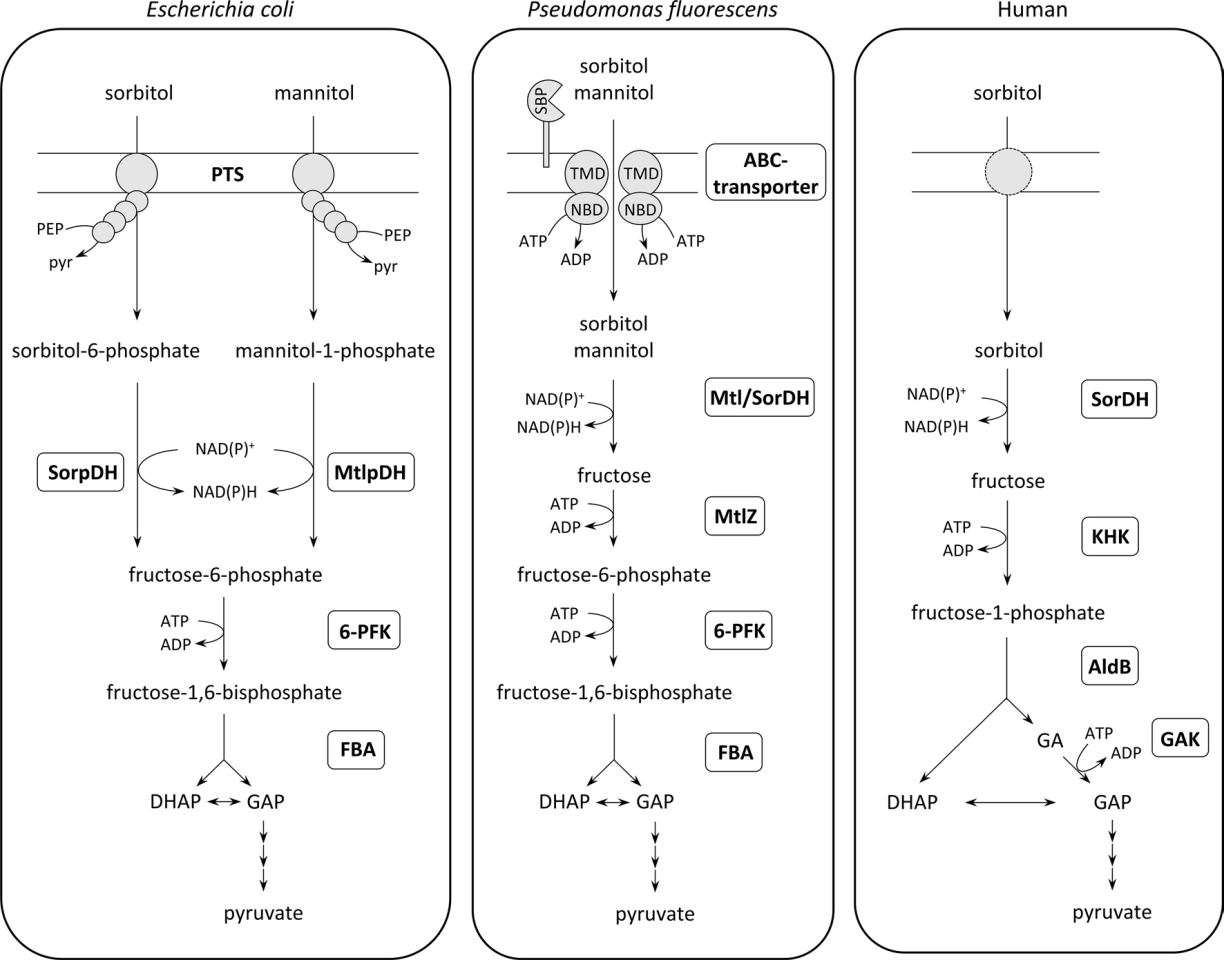
Proposed pathways of uptake and degradation of mannitol and sorbitol in the bacteria *Escherichia coli* and *Pseudomonas fluorescens* and in human. PTS, phosphoenolpyruvate-dependent phosphotransferase system; SorpDH, sorbitol-6-phosphate dehydrogenase; MtlpDH, mannitol-6-phosphate dehydrogenase; 6-PFK, fructose-6-phosphate kinase; FBA, fructose-1,6-bisphosphate aldolase; SBP, substrate-binding protein; TMD, transmembrane domain; NBD, nucleotide-binding domain; Mtl/SorDH, bifunctional mannitol/sorbitol dehydrogenase; MtlZ, fructokinase; SorDH, sorbitol dehydrogenase; KHK, ketohexokinase; AldB, aldolase B; GAK, glycerate kinase; PEP, phosphoenolpyruvate; pyr, pyruvate; DHAP, dihydroxyacetone phosphate; GAP, glyceraldehyde-3-phosphate; GA, glyceraldehyde

For Bacteria, e.g. *E. coli* and *Pseudomonas fluorescens,* different mechanisms of uptake and degradation of sugar alcohols have been described. In *E. coli*, uptake of sorbitol and mannitol is catalyzed by a single PTS forming sorbitol-6-phosphate and mannitol-1-phosphate, respectively (Lengeler 1975). These phosphorylated sugar alcohols are each oxidized by specific dehydrogenases to fructose-6-phosphate (F6P), followed by phosphorylation of F6P to FBP by fructose-6-phosphate kinase (6-PFK). FBP is cleaved via FBA to form triosephosphates that are degraded to pyruvate (Fig. 1) (Novotny et al. 1984; Chase 1986; Mayer and Boos 2005).

In contrast, both sorbitol and mannitol are taken up by a single ABC transporter in *P. fluorescens* and the subsequent oxidation of both alcohols to fructose involves a promiscuous dehydrogenase. Fructose is then phosphorylated by fructokinase to F6P that is further converted to triosephosphates and pyruvate by enzymes of the classical EM pathway (Fig. 1) (Brünker et al. 1997, 1998).

In human, the uptake of sorbitol—by a mechanism not yet defined—yields intracellular sorbitol that is converted to fructose by sorbitol dehydrogenase and phosphorylated to F1P via ketohexokinase (KHK) (Maret and Auld 1988; Tran 2017). F1P is cleaved by aldolase B to glyceraldehyde and dihydroxyacetone phosphate (DHAP), which are both converted to glyceraldehyde-3-phosphate by glyceraldehyde kinase and DHAP isomerase, and further degraded to pyruvate (Fig. 1).

Here, we present a comprehensive analysis of the uptake and degradation pathways of the sugar alcohols mannitol and sorbitol in the haloarchaeon *Haloarcula hispanica*. It is shown that the sugar alcohols are taken up by an ABC transporter and further oxidized to fructose by distinct dehydrogenases. Phosphorylation of fructose to F1P is catalyzed by a haloarchaeal KHK, which is the first proof of an in vivo function of a KHK in the metabolism of prokaryotes. Further conversion of F1P to triosephosphates involves 1-PFK and a Class I FBA. Finally, two different transcriptional regulators have been identified that are involved in regulation of uptake and degradation of mannitol and sorbitol.

## Materials and methods

### Growth experiments

*Haloarcula hispanica* DF60 and mutant strains were grown aerobically in synthetic medium at 37 °C (Dambeck and Soppa 2008; Sutter et al. 2016). Growth was followed over time as optical density at 600 nm. The medium contained either 1% casamino acids, 10 mM D-glucose, D-fructose, D-mannitol or D-sorbitol as carbon and energy source and was supplemented with 50 μg/ml uracil. Mutants were pregrown on 1% casamino acids, except Δ*fbaB1* that was pregrown on 10 mM glucose.

The Δ*fba* mutant of *H. volcanii* H26 (Pickl et al. 2012) was used for complementation with *fbaB1* of *H. hispanica*. For this experiment the *H. hispanica fbaB1* gene was cloned into pTA963. By this strategy the *fbaB1* gene is under control of the p.*tnaA* promotor, and expression of FBA can be induced by the addition of tryptophane. The generated plasmid was used for transformation of the *H. volcanii* Δ*fba* mutant. Growth experiments were performed in synthetic medium containing 10 mM fructose and 0.1 or 1.0 mM tryptophane at 42 °C without the addition of uracil.

### Generation of deletion mutants

Deletion mutants of *H. hispanica* DF60 were generated following the principles of the pop-in/pop-out strategy described for *H. volcanii* and for *H. hispanica* (Bitan-Banin et al. 2003; Allers et al. 2004; Allers and Mevarech 2005; Liu et al. 2011; Johnsen et al. 2020). The uracil auxotrophic *H. hispanica* strain DF60 lacks the *pyrF* gene. For pop-in and pop-out a modified version of the suicide vector pTA131 (Allers et al. 2004) was used. Instead of the *pyrE2* gene of pTA131, the modified vector pTA131-HAH-*pyrF* carries the *pyrF* gene of *H. hispanica* under the control of its native promotor, which allows selection and counterselection in DF60. Deletion mutants of the following genes were generated: HAH_5138 (*mscS*), HAH_5147 (*mscM*), HAH_1078 (*khk*), HAH_1077 (*fruK*), HAH_1079 (*fbaB1*), HAH_5148 (*mscR*), HAH_5146 (*mscE*), and HAH_1560 (*gfcR*).

### Overexpression and purification of enzymes

Genes encoding putative sorbitol dehydrogenase (*mscS*; HAH_5138), mannitol dehydrogenase (*mscM*; HAH_5147), fructose-1-phosphate kinase (*fruK*; HAH_1077), and fructose-1,6-bisphosphate aldolase (*fbaB1*; HAH_1079) from *H. hispanica*, and the ketohexokinase gene (*khk*; rrnAC0343) from *Haloarcula marismortui* were amplified from genomic DNA and cloned into vector pTA963 according to the strategy of Allers et al. (2010). The 5´ends of the genes were fused with a 6 × CAC encoding an N-terminal 6 × His tag. After cloning and multiplication in *E. coli* XL1-Blue mrf´, plasmids were used for transformation of *H. volcanii* strain H1209 and overexpression was performed in complex medium (Allers 2010; Pickl et al. 2012).

For purification of recombinant proteins, cell pellets were suspended in buffer (100 mM Tris–HCl, 1.5 M KCl, 5 mM imidazole, pH 8.2) and cells were disrupted using a French pressure cell press (16,000 PSI) followed by centrifugation at 46,000×*g*. Purification was performed using Ni–NTA-affinity and size exclusion chromatography as described (Kuprat et al. 2021). Determination of protein concentration was performed by the method of Bradford using bovine serum albumin fraction V as standard, and purity of recombinant protein was checked by SDS PAGE. Native molecular masses were determined with a size exclusion column (superdex HiLoad 16/600 200 pg; GE Healtcare) that was calibrated with proteins from the HMW and LMW kits (GE Healthcare).

### Characterization of enzymes

Enzyme activity was measured photometrically at 37 °C in assay mixtures of 1 ml total volume. Kinetic parameters, *V*_max_ and *K*_M_, and standard errors were calculated with the Origin2017 software according to the Michaelis–Menten equation.

**Mannitol dehydrogenase** (mannitol + NAD^+^  ⇌ fructose + NADH) was measured in the direction of mannitol oxidation in an assay mixture containing 50 mM Tris–HCl, 1.5 M KCl (pH 7.5), 1 mM MnCl_2_, 1 mM NAD^+^, and 10 mM mannitol. Oxidation of sorbitol was tested at up to 100 mM. In the direction of fructose reduction, the assay contained 50 mM Tris–HCl, 1.5 M KCl (pH 7.5), 1 mM MnCl_2_, 0.3 mM NADH, and 10 mM fructose.

**Sorbitol dehydrogenase** (sorbitol + NAD^+^  ⇌ fructose + NADH) was tested in the direction of sorbitol oxidation using an assay mixture containing 50 mM Tris–HCl, 1.5 M KCl (pH 7.5), 1 mM MnCl_2_, 2 mM NAD^+^, and 15 mM sorbitol. Oxidation of mannitol was assayed at up to 100 mM. In the direction of fructose reduction, the assay mixture contained 50 mM Tris–HCl, 1.5 M KCl (pH 7.5), 1 mM MnCl_2_, 0.3 mM NADH, and up to 150 mM fructose.

**Ketohexokinase** (fructose + ATP → fructose-1-phosphate + ADP) activity was tested according to Ortjohann and Schönheit (2024). The assay mixture contained 100 mM Tris–HCl, 1.5 M KCl (pH 7.5), 20 mM fructose, 5 mM ATP, 10 mM MgCl_2_, 2.5 mM phosphoenolpyruvate, 0.3 mM NADH, 11 U pyruvate kinase, and 5 U lactate dehydrogenase. KCl optimum was determined between 0 M and 3.5 M. Activity was also tested with D-glucose, D-galactose, L-rhamnose, D-xylose, L-arabinose, and D-ribose at 5 mM and at 20 mM each.

**Fructose-1-phosphate kinase** (fructose-1-phosphate + ATP → fructose-1,6-bisphosphate + ADP) was measured at pH 7.5 in 50 mM Tris–HCl, 1.5 M KCl containing 4 mM fructose-1-phosphate, 5 mM ATP, 10 mM MgCl_2_, 2.5 mM phosphoenolpyruvate, 0.3 mM NADH, 4 U pyruvate kinase, and 11 U lactate dehydrogenase. Phosphorylation of fructose-6-phosphate was tested with up to 35 mM.

### Sequence and phylogenetic analyses

Amino acid sequence alignments were calculated with Clustal X 2.1 (Larkin et al. 2007), and structure-based sequence alignments were performed with Expresso (Armougom et al. 2006). Alignments were depicted with ESPript 3.0 (Robert and Gouet 2014). Phylogenetic trees were calculated on the basis of multiple amino acid sequence alignments using the neighbor-joining algorithm and depicted with the software MEGAX (Kumar et al. 2018).

## Results and discussion

Here, we present a pathway for mannitol and sorbitol degradation in the haloarchaeon *Haloarcula hispanica*.

### Haloarcula hispanica grows on mannitol and sorbitol

**Fig. 2:**
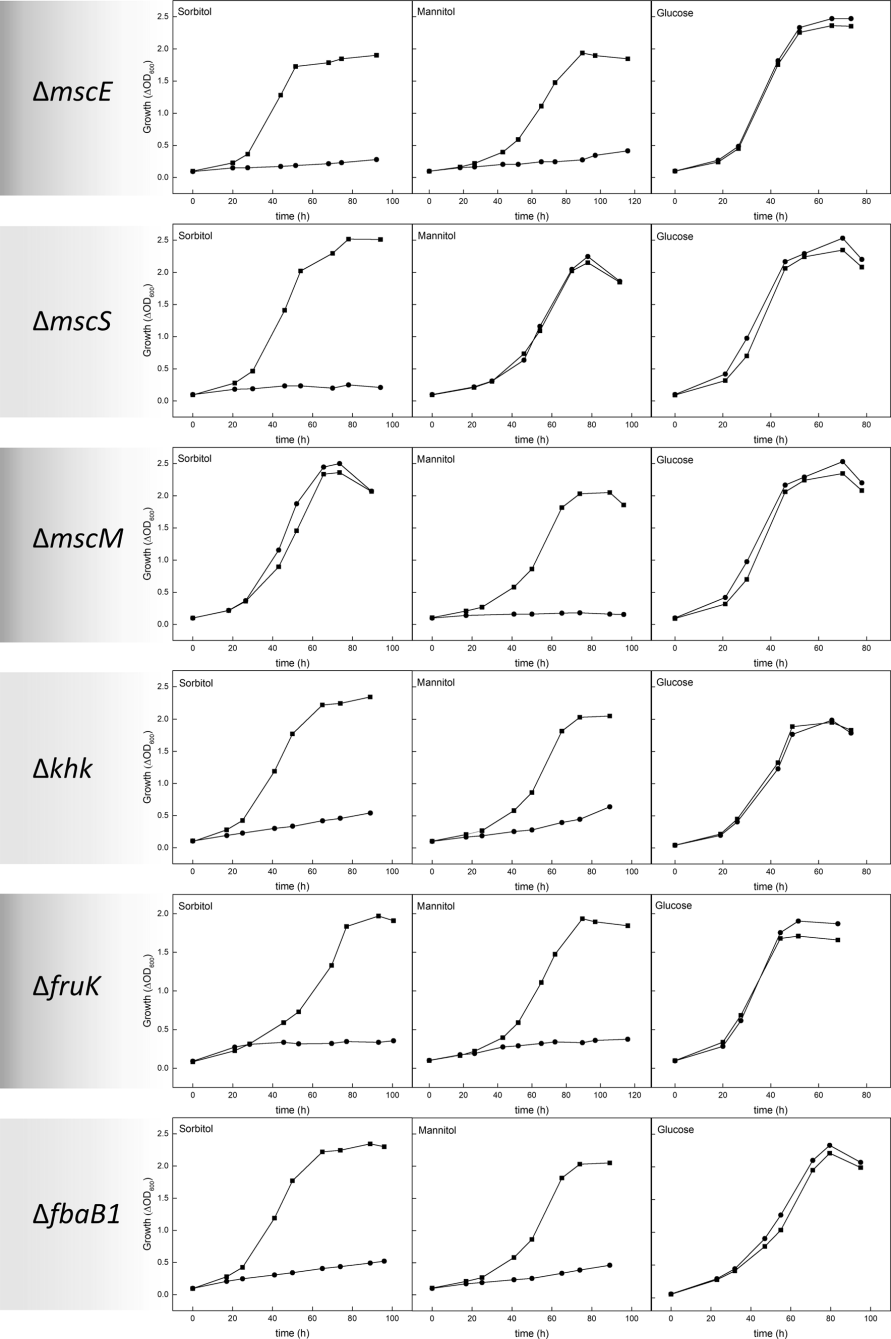
Growth analyses of deletion mutants of genes involved in the degradation of sorbitol and mannitol in *H. hispanica*. Growth of deletion mutant strains (●) Δ*mscE*, Δ*mscS*, Δ*mscM*, Δ*khk*, Δ*fruK,* and Δ*fbaB1* was performed on 10 mM each of sorbitol, mannitol, and glucose, respectively, in comparison to the wild-type strain (■)

*Haloarcula hispanica* DF60 is able to grow on mannitol and sorbitol as sole carbon and energy sources (Fig. 2). With 10 mM mannitol or sorbitol, the cells grew with doubling times of 15–17 h and 11–12 h, respectively, up to cell densities at 600 nm of 2.0–2.5. Growth on mannitol has previously been reported for related *Haloarcula vallismortis* (Altekar and Rangaswamy 1992). Recently, growth of *H. volcanii* on mannitol has been excluded (Ortjohann and Schönheit 2024).

### Uptake of sorbitol and mannitol via a CUT1 type ABC transporter

**Fig. 3:**
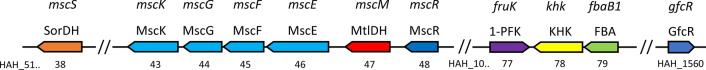
Genomic organization of genes involved in uptake and degradation of sorbitol and mannitol in *Haloarcula hispanica*. Mannitol and sorbitol catabolism, *msc* cluster: Genes of sugar alcohol uptake encode an ABC transporter composed of a substrate-binding protein (MscE, *mscE*), two transmembrane domains (MscF/MscG, *mscF*/*mscG*), and a nucleotide-binding domain (MscK, *mscK*); genes of sorbitol and mannitol oxidation encode a sorbitol dehydrogenase (SorDH, *mscS*) and mannitol dehydrogenase (MtlDH, *mscM*). Fructose cluster: ketohexokinase (KHK, *khk*), fructose-1-phosphate kinase (1-PFK, *fruK*) and fructose-1,6-bisphosphate aldolase (FBA, *fbaB1*). Two transcriptional regulators (MscR/GfcR, *mscR*/*gfcR*) are involved in the activation of genes involved in sorbitol and mannitol degradation

In the genome of *H. hispanica*, a substrate-binding protein (SBP) was identified, which is part of a putative ABC transporter gene cluster and shares 42% sequence identity with the SBP of the polyol ABC transporter from *Pseudomonas fluorescens* (Brünker et al. 1998). Besides the SBP-encoding *mscE* gene (HAH_5146), this cluster encodes two transmembrane domains (TMDs; *mscF*, *mscG*; HAH_5145, HAH_5144) and one nucleotide-binding domain (NBD; *mscK*, HAH_5143). Directly up- and downstream of the ABC gene cluster, two dehydrogenase genes and one gene encoding a transcriptional regulator were identified. Together, these genes form an operon that is likely involved in mannitol and sorbitol degradation (Fig. 3). We designated this gene cluster as mannitol and sorbitol catabolism (*msc*) cluster, comprising the genes *mscSKGFEMR*.

To test the functional involvement of the ABC transporter in the uptake of mannitol and sorbitol, a deletion mutant of the SBP-encoding gene *mscE* was constructed. This mutant was unable to grow on sugar alcohols, while growth on glucose was not affected (Fig. 2), suggesting a role for MscE as SBP of an ABC transporter that is promiscuous for the uptake of both mannitol and sorbitol.

**Fig. 4:**
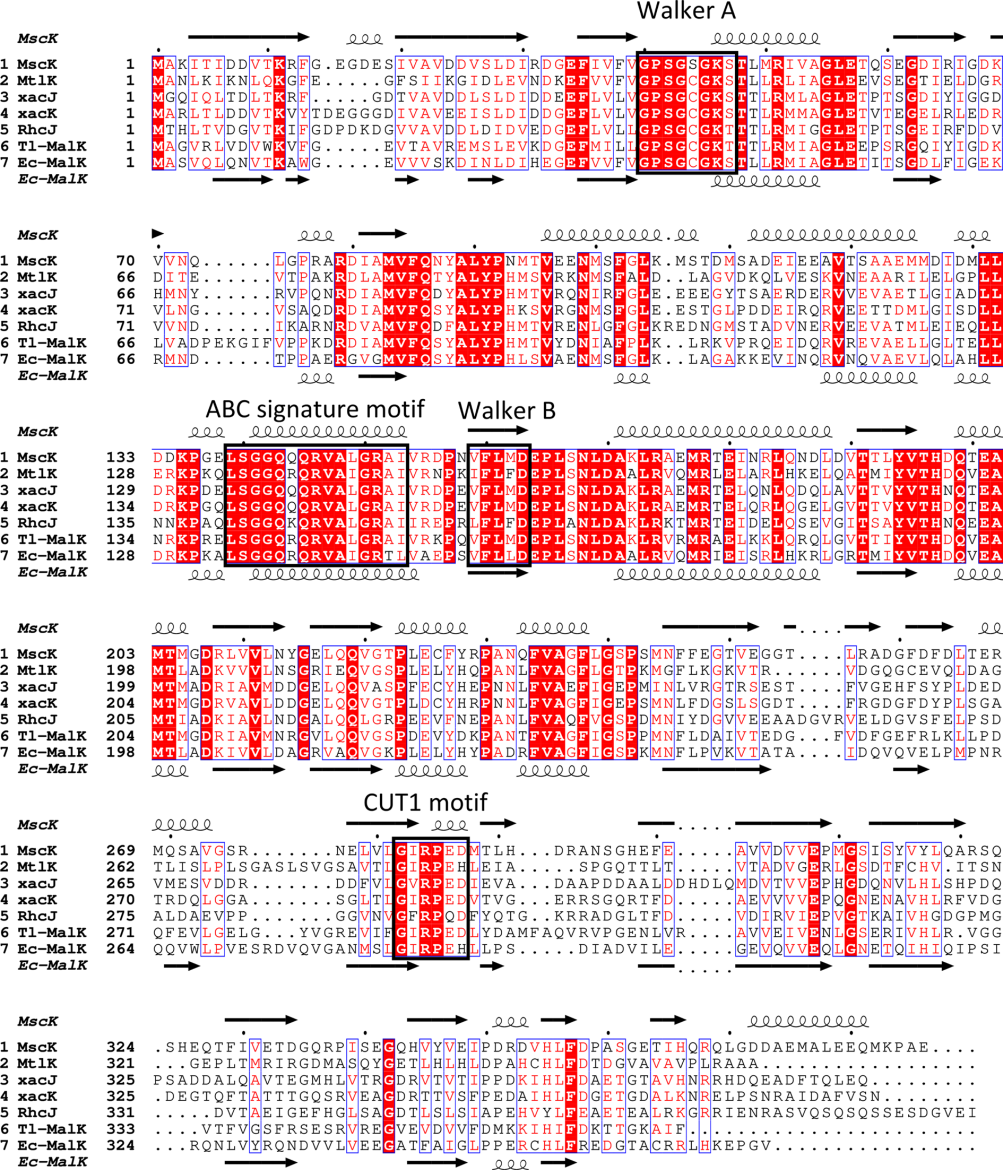
Multiple amino acid sequence alignment of MscK from *H. hispanica* and NBDs of the CUT1 family of ABC transporters. MscK is shown together with sequences of the NBDs of the polyol ABC transporter from *Pseudomonas fluorescens* (MtlK) (Brünker et al. 1998), of the pentose ABC transporter (XacJ/XacK) (Johnsen et al. 2019) and the rhamnose ABC transporter (RhcJ) from *H. volcanii* (Reinhardt et al. 2019), the trehalose/maltose ABC transporter from *Thermococcus litoralis* (Tl-MalK) (Diederichs et al. 2000), and the maltose/maltodextrin ABC transporter from *E. coli* (Ec-MalK) (Gilson et al. 1982). MscK contains the typical ATPase features Walker A and Walker B, the ABC signature motif (PROSITE: PS00211), and the CUT1 signature motif. Crystal structure-based secondary structure of MalK from *E. coli* (PDB: 1Q12) is in good agreement with the secondary structure of MscK derived from AlphaFold prediction (AF-G0I055-F1-model_v4). UniProt accession: MscK, G0I055; MtlK, O30494; XacJ, D4GP38; XacK, D4GP39; RhcJ, D4GPB1; Tl-MalK, Q9YGA6; P68187

Sequence analysis of the NBD MscK classifies the ABC transporter for mannitol/sorbitol in *H. hispanica* as a member of the CUT1 transporter family (carbohydrate uptake transporter class 1) (Schneider 2001; Zheng et al. 2013). A multiple amino acid sequence alignment of MscK and NBDs of the polyol ABC transporter of *P. fluorescens*, and selected CUT1 transporters from Archaea, including the recently identified haloarchaeal pentose ABC transporter (Johnsen et al. 2019), and MalK from *E. coli* is given in Fig. 4. A typical CUT1 transporter is encoded by four genes and composed of SBP, two TMDs forming a heterodimer and two NBDs forming a homodimer (Schneider 2001), which is in accordance with the four genes *mscEFGK* analyzed here. Further characteristic features of the CUT1 family include the ATP binding Walker A and B sites, the classical ABC transporter signature, and the CUT1 specific sequence motif G[IV]RPE[DH], which are indicated in Fig. 4. In Archaea, CUT1 ABC transporter represent the dominant transport system for the uptake of carbohydrates. Only one CUT2 ABC transporter has been characterized in Archaea so far, the D-xylose/L-arabinose transporter in *Sulfolobus acidocaldarius* (Wagner et al. 2018). It is interesting to note that members of the peptide/opine/nickel uptake transporter (PepT) transporter family have also been shown to be involved in the transport of sugars, i.e. the ABC transporter for galactose in *H. volcanii* (Tästensen et al. 2020).

### Mannitol and sorbitol are oxidized to fructose via two distinct dehydrogenases

The *msc* cluster contains two genes, *mscS* (HAH_5138) and *mscM* (HAH_5147)*,* that encode putative dehydrogenases. The functional involvement in degradation of sugar alcohols was analyzed with the mutants Δ*mscS* and Δ*mscM*, and the encoded enzymes SorDH and MtlDH were characterized. The mutant Δ*mscS* was not able to grow on sorbitol, but growth on mannitol was not affected (Fig. 2). Conversely, Δ*mscM* did not grow on mannitol, whereas growth on sorbitol was not affected (Fig. 2). These results indicate that *mscS* and *mscM* encode functionally distinct dehydrogenases, SorDH and MtlDH.

**Fig. 5:**
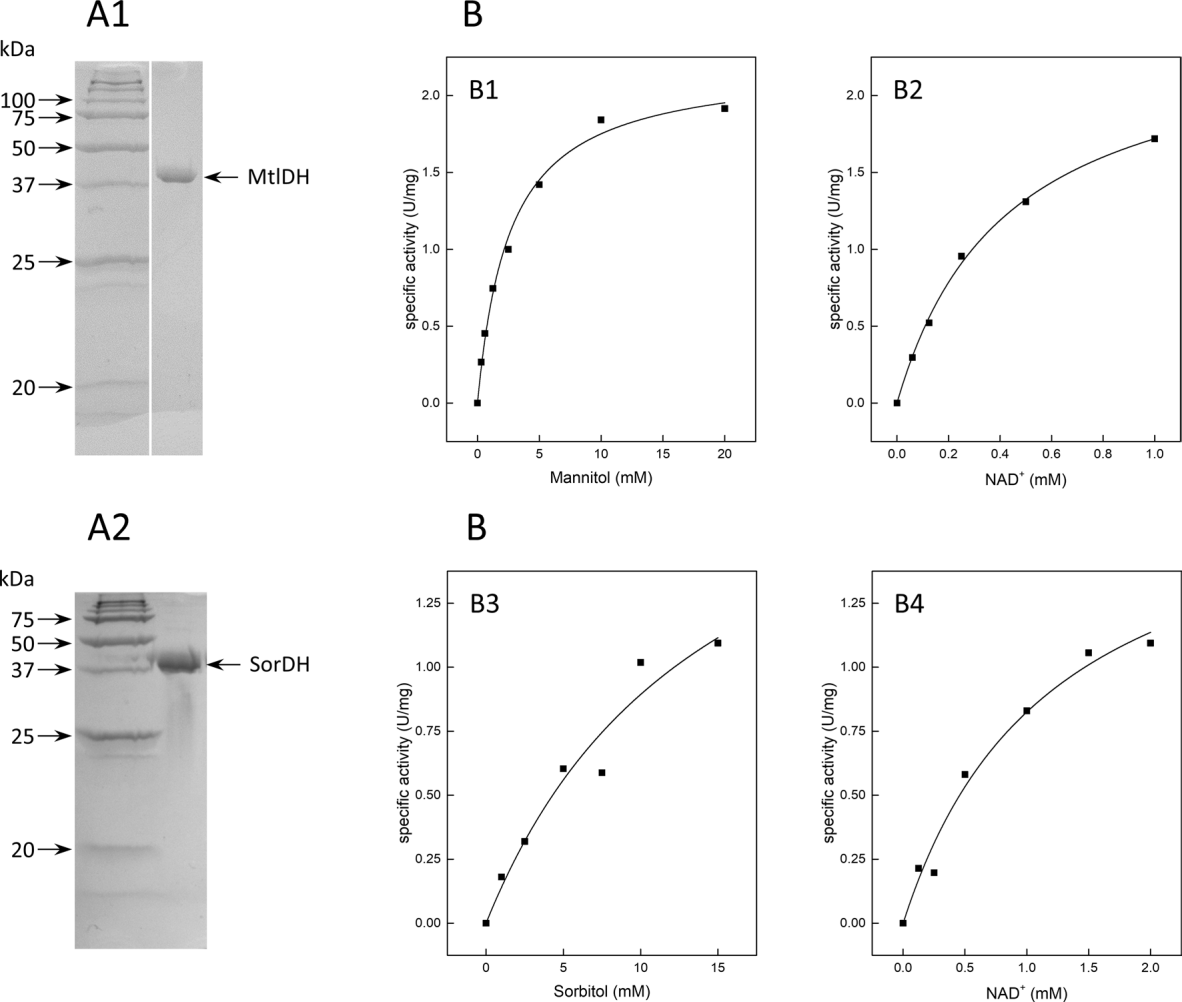
Purification and kinetic parameters of mannitol dehydrogenase (MtlDH) and sorbitol dehydrogenase (SorDH) from *H. hispanica*. **A** SDS-PAGE of purified His-tagged MtlDH (A1) and SorDH (A2)*.* Arrows on the left side indicate molecular mass standards (kDa). **B** Rate dependence of MtlDH on mannitol (B1) and NAD^+^ (B2) and of SorDH on sorbitol (B3) and NAD^+^ (B4), respectively

The recombinant enzymes MtlDH (*mscM*) and SorDH (*mscS*) were overexpressed in *H. volcanii* and purified to apparent homogeneity by affinity and size exclusion chromatography (Fig. 5A1 & A2). MtlDH catalyzed the NAD^+^-dependent oxidation of mannitol with a *V*_max_ of 2.2 ± 0.07 U/mg and *K*_M_ values of 2.6 ± 0.27 mM for mannitol and 0.42 ± 0.03 mM for NAD^+^ (Fig. 5B); NADP^+^ was not accepted. MtlDH catalyzed the oxidation of sorbitol with a *V*_max_ of 0.2 ± 0.007 U/mg and a *K*_M_ value of 27.6 ± 2.66 mM, i.e. at a 120-fold lower catalytic efficiency for sorbitol, indicating that MtlDH is highly specific for mannitol. Reduction of fructose was catalyzed with a *V*_max_ of 24 ± 1.09 U/mg and a *K*_M_ value of 73 ± 6.78 mM.

SorDH catalyzed the NAD^+^-dependent oxidation of sorbitol with a *V*_max_ of 2.2 ± 0.71 U/mg and *K*_M_ values of 14.7 ± 7.8 mM for sorbitol and 1.2 ± 0.35 mM for NAD^+^ (Fig. 5B); NAD^+^ could not be replaced by NADP^+^. Mannitol was oxidized at a 70-fold lower catalytic efficiency, showing a *V*_max_ of 0.27 ± 0.076 U/mg and a *K*_M_ value of 124 ± 62.8 mM, characterizing the enzyme as sorbitol dehydrogenase. The reverse reaction, the reduction of fructose by NADH, was catalyzed with *V*_max_ and *K*_M_ values for fructose of 4.2 ± 1.7 U/mg and 279 ± 158 mM, respectively.

**Fig. 6:**
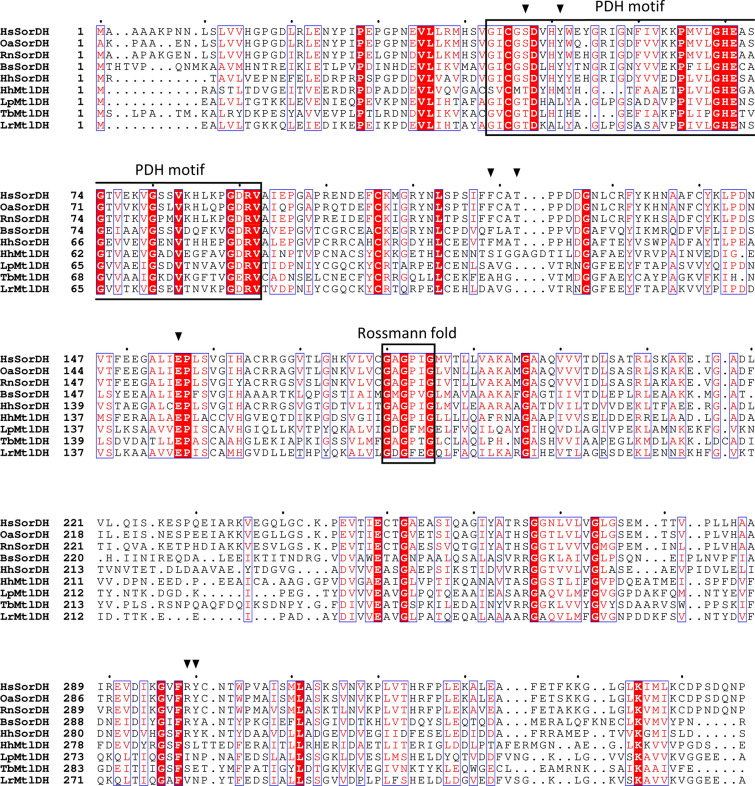
Structure-based multiple amino acid sequence alignment of sorbitol dehydrogenase and mannitol dehydrogenase from *H. hispanica* (HhSorDH/HhMtlDH) and of sorbitol and mannitol dehydrogenases of the MDR superfamily. HhSorDH and HhMtlDH are shown together with sorbitol dehydrogenase from human (HsSorDH) (Pauly et al. 2003), from *Ovis aries* (OaSorDH) (Yennawar et al. 2011), from *Rattus norvegicus* (RnSorDH) (Johansson et al. 2001), and from *Bacillus subtilis* (BsSorDH) (Ng et al. 1992) and with mannitol dehydrogenase from *Tuber borchi* (TbMtlDH) (Ceccaroli et al. 2007), from *Leuconostoc pseudomesenteroides* (LpMtlDH) (Hahn et al. 2003), and from *Lactobacillus reuteri* (LrMtlDH) (Sasaki et al. 2005). The positions of the PDH motif (Nordling et al. 2002) and the central part of the Rossmann fold (Wierenga et al. 1986) are marked with boxes. Amino acid residues that have been proposed to be involved in binding of sorbitol in RnSorDH are conserved in HhSorDH (▼). UniProt accession: HsSorDH, Q00796; OaSorDH, P07846; RnSorDH, P27867; BsSorDH, Q06004; LrMtlDH, Q6ECH5; LpMtlDH, Q83VI5; TbMtlDH, Q1ACW3; HhSorDH, G0I050; HhMtlDH, G0I059

Sequence analyses of MtlDH and SorDH from *H. hispanica* assigned these enzymes to the polyol dehydrogenase (PDH) family of the medium-chain dehydrogenase (MDR) superfamily (Nordling et al. 2002; Riveros-Rosas et al. 2003; Persson et al. 2008). In accordance, the characteristic PDH motif ([GA]-[VIL]-[CS]-[GN]-[STA]-D-[VILMS]-[HKP]-x(14,27)-G-H-[ED]-x(2)-G-x-[VI]-x(10,12)-G-[DEQ]-x-[IV]) (Nordling et al. 2002) is present in SorDH and, with few deviations, in MtlDH of *H. hispanica* (Fig. 6). A structure-based multiple amino acid sequence alignment of mannitol and sorbitol dehydrogenases from *H. hispanica* and from Bacteria and Eukarya is shown in Fig. 6. On the basis of the crystal structure of sorbitol dehydrogenase from *Rattus norvegicus* (RnSorDH) with modeled sorbitol, amino acid residues have been postulated to be involved in substrate binding, namely Glu156, Arg299, Ser47, Tyr51, Phe119, Thr122, and Tyr300 (Johansson et al. 2001). All corresponding positions are identical in SorDH of *H. hispanica* suggesting an involvement of the equivalent amino acid residues in binding of sorbitol (Fig. 6). Sequences of SorDH and MtlDH also comprise the glycine rich central part of the Rossmann fold sequence motif, G-x-G-x-x-G (Fig. 6), which is involved in binding of coenzymes NAD(P)^+^ (Wierenga et al. 1986; Geertz-Hansen et al. 2014).

**Fig. 7:**
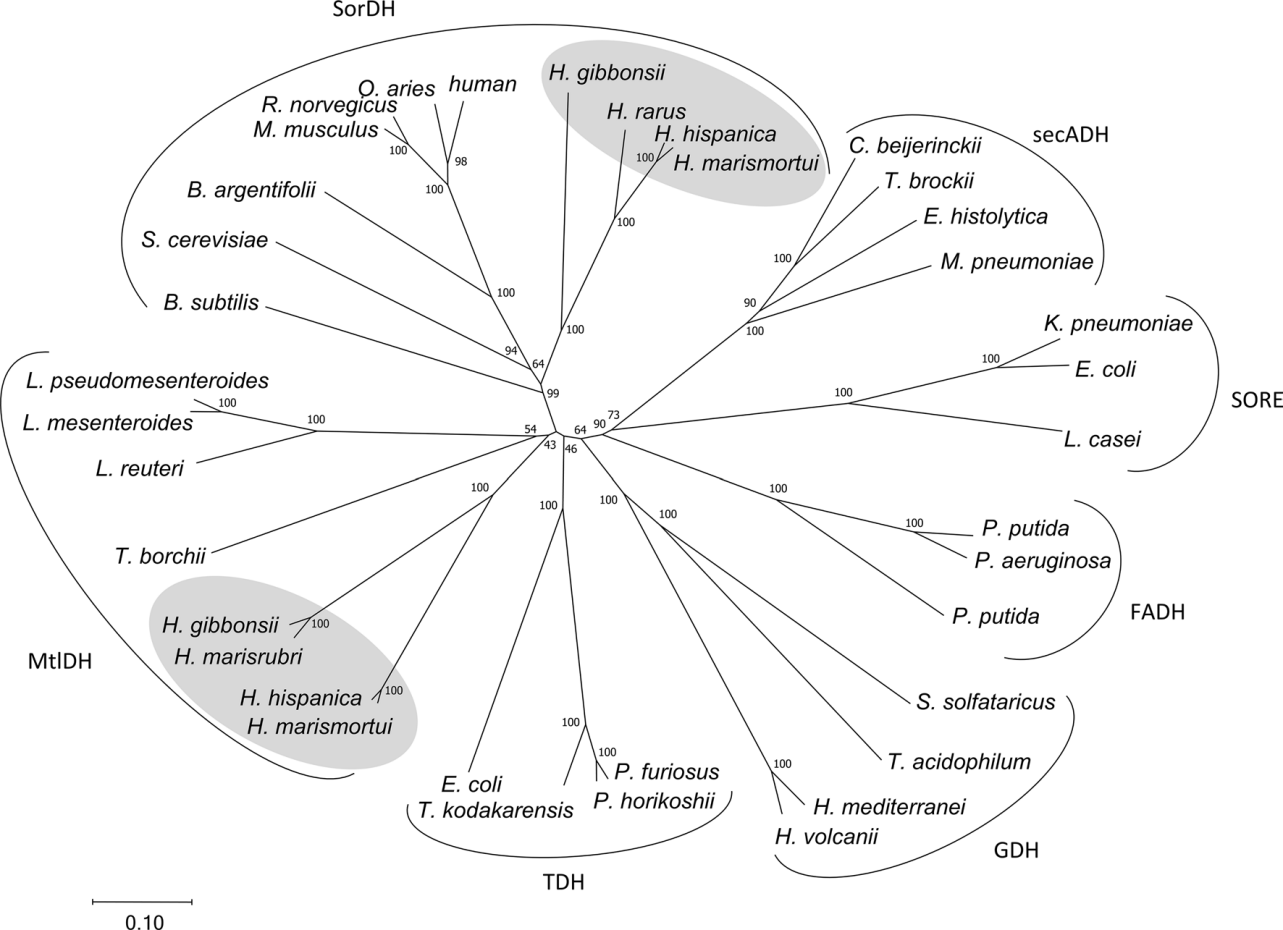
Phylogenetic relation of sorbitol and mannitol dehydrogenase from *H. hispanica* and members of polyol dehydrogenase (PDH) subfamilies of the medium chain dehydrogenase (MDR) superfamily. Subfamilies within the PDH family form distinct clades. Sorbitol dehydrogenase (SorDH) and mannitol dehydrogenase (MtlDH) from *H. hispanica* cluster together with homologs from other haloarchaea (highlighted in grey) and form separate branches together with characterized SorDHs and MtlDHs. SorDH: sorbitol dehydrogenase; MtlDH: mannitol dehydrogenase; TDH: threonine dehydrogenase; secADH: secondary alcohol dehydrogenase; SORE: L-sorbose-1-phosphate reductase; GDH: archaeal glucose dehydrogenase; FADH: formaldehyde dehydrogenase. UniProt accession: SorDH: *B. subtilis*, Q06004; *S. cerevisiae*, P35497; *H. hispanica*, G0I050; *B. argentifolii*, O96496; *M. musculus*, Q64442; *R. norvegicus*, P27867; *O. aries*, P07846; *H. sapiens*, Q00796; *H. gibbonsii*, M0HN94; *H. rarus*, WP_256422950.1; *H. marismortui*, Q5V6U8; MtlDH: *H. hispanica*, G0I059; *H. marismortui*, Q5V6V7; *T. borchii*, Q1ACW3; *L. mesenteroides*, Q8KQG6; *L. pseudomesenteroides*, Q83VI5; *L. reuteri*, Q6ECH5; *H. gibbonsii*, M0H624; *H. marisrubri*, A0A2P4NQW5; TDH: *E. coli*, P07913; *P. furiosus*, Q8U259; *T. kodakarensis*, Q5JI69; *P. horikoshii*, O58389; secADH: *C. beijerinckii*, P25984; *E. histolytica*, P35630; *T. brockii*, P14941; *M. pneumoniae*, P75214; SORE: *K. pneumoniae*, P37084; *L. casei*, Q9RGG8; *E. coli* A0AAE6DZQ2; GDH: *H. volcanii*, D4GVT2; *H. mediterranei*, Q977U7; *T. acidophilum*, P13203; *S. solfataricus*, O93715; FADH: *P. putida*, P4615; *P. aeruginosa*, Q9HTE3; *P. putida*, Q52078

Besides sorbitol and mannitol dehydrogenases (Riveros-Rosas et al. 2003; Ceccaroli et al. 2007), the PDH family contains various different dehydrogenase subfamilies, such as archaeal glucose dehydrogenase (GDH), secondary alcohol dehydrogenase (secADH), L-sorbose-1-phosphate reductase, formaldehyde dehydrogenase (FADH), and threonine dehydrogenase (TDH). Phylogenetic analyses showed that these PDH families form separate branches (Fig. 7). SorDH and MtlDH of *H. hispanica* analyzed in this study and haloarchaeal homologs cluster together with sorbitol and mannitol dehydrogenases from Eukarya and Bacteria (Fig. 7), indicating that these sugar alcohol dehydrogenases cluster according to their enzymatic function rather than to their phylogenetic affiliation.

### Ketohexokinase

Three genes have been identified in *H. hispanica* involved in the degradation of fructose, which is formed as an intermediate in the oxidation of sorbitol and mannitol. These genes form a cluster (HAH_1077 to HAH_1079) and encode ketohexokinase, fructose-1-phosphate kinase, and fructose-1,6-bisphosphate aldolase (Fig. 3). KHK from *H. hispanica* (HAH_1078) shows 53% sequence identity to the recently identified haloarchaeal KHK from *Haloferax volcanii* that has been shown to catalyze the ATP-dependent phosphorylation of fructose to fructose-1-phosphate (Ortjohann and Schönheit 2024). A *khk* deletion mutant of *H. hispanica* was constructed and analyzed in growth experiments on mannitol and sorbitol. Growth of the mutant on both sugar alcohols was almost completely abolished as compared to the wild-type, while growth on glucose was unaffected (Fig. 2). These findings indicate that KHK from *H. hispanica* has a function in the degradation of sugar alcohols.

**Fig. 8:**
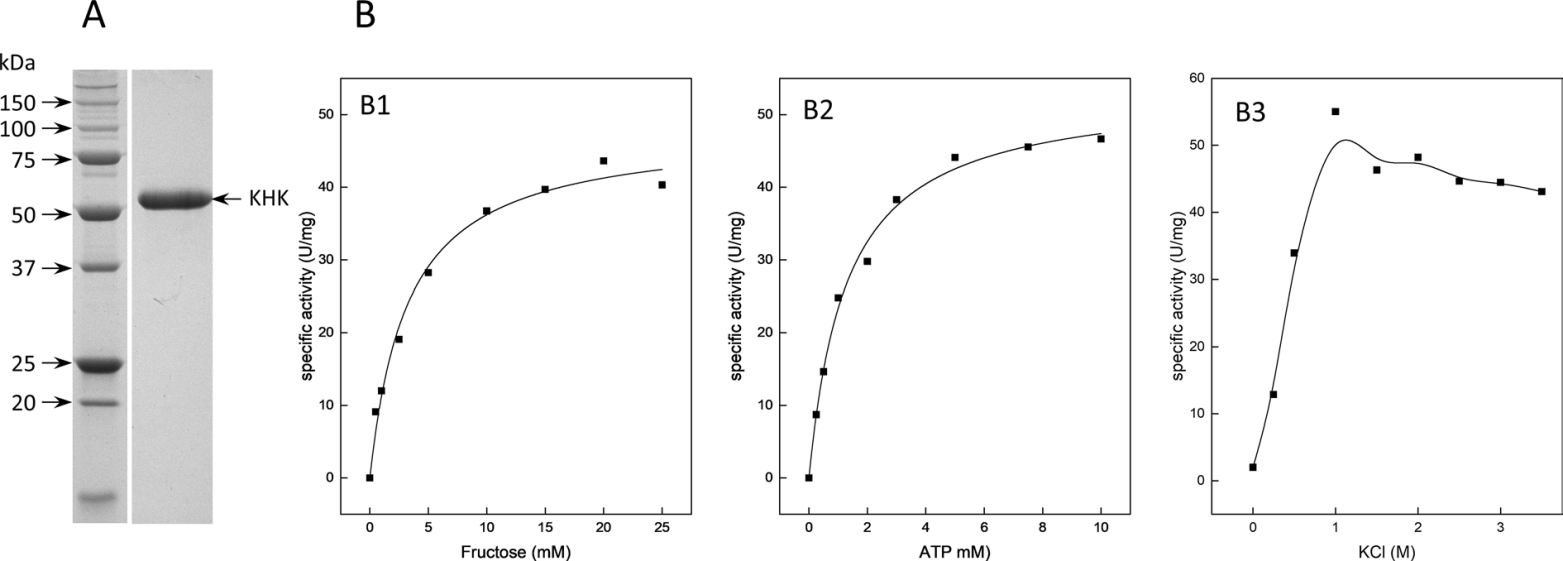
Purification and kinetic parameters of ketohexokinase (KHK) from *H. marismortui*. **A** SDS-PAGE of purified His-tagged KHK*.* Arrows on the left side indicate molecular mass standards (kDa). **B** Rate dependence of KHK on fructose (B1), ATP (B2), and KCl (B3)

KHK from *H. hispanica* shows 99% sequence identity to the homologous sequence in *Haloarcula marismortui*; the enzyme of *H. marismortui* was overexpressed in *H. volcanii* and purified by affinity and size exclusion chromatography (Fig. 8A). The enzyme catalyzed the ATP-dependent phosphorylation of fructose with a *V*_max_ of 47.9 ± 1.76 U/mg and *K*_M_ values of 3.25 ± 0.45 and 1.29 ± 0.12 mM for fructose and ATP, respectively. The enzyme was specific for fructose and did not catalyze the phosphorylation of glucose, galactose, rhamnose, xylose, arabinose, or ribose. KHK was most active (100%) at 1 M KCl and showed no activity at 0 M KCl, while 85% activity could be determined at 3.5 M KCl (Fig. 8B). KHK from *H. marismortui* has a calculated molecular mass of 40.2 kDa and showed a native molecular weight of 87 kDa according to size exclusion chromatography, indicating a homodimeric oligomerization of the subunits as has been described for KHK from *H. volcanii* (Ortjohann and Schönheit 2024). Haloarchaeal KHKs are only distantly related to KHKs from Eukarya and have been shown to form a distinct phylogenetic cluster within the ribokinase/pfkB superfamily (Ortjohann and Schönheit 2024).

While the in vivo function of KHK in *H. volcanii* is not known, we demonstrate here the essential function of KHK in *H. hispanica* in the degradation of mannitol and sorbitol. KHK phosphorylates the intermediate of mannitol and sorbitol oxidation, fructose, to F1P. This is the first report of a physiological in vivo function of a KHK in prokaryotes.

### Fructose-1-phosphate kinase

In *H. hispanica*, the *fruK* gene HAH_1077 encodes a putative fructose-1-phosphate kinase, 1-PFK, which shares 54% sequence identity with the characterized 1-PFK from *H. volcanii* that is involved in fructose degradation (Pickl et al. 2012). In *H. volcanii*, 1-PFK catalyzes the ATP-dependent phosphorylation of fructose-1-phosphate, which is formed from fructose via uptake by a PTS, to fructose-1,6-bisphosphate (FBP). The functional involvement of 1-PFK from *H. hispanica* in degradation of sugar alcohols was analyzed with a deletion mutant followed by characterization of the recombinant enzyme.

**Fig. 9:**
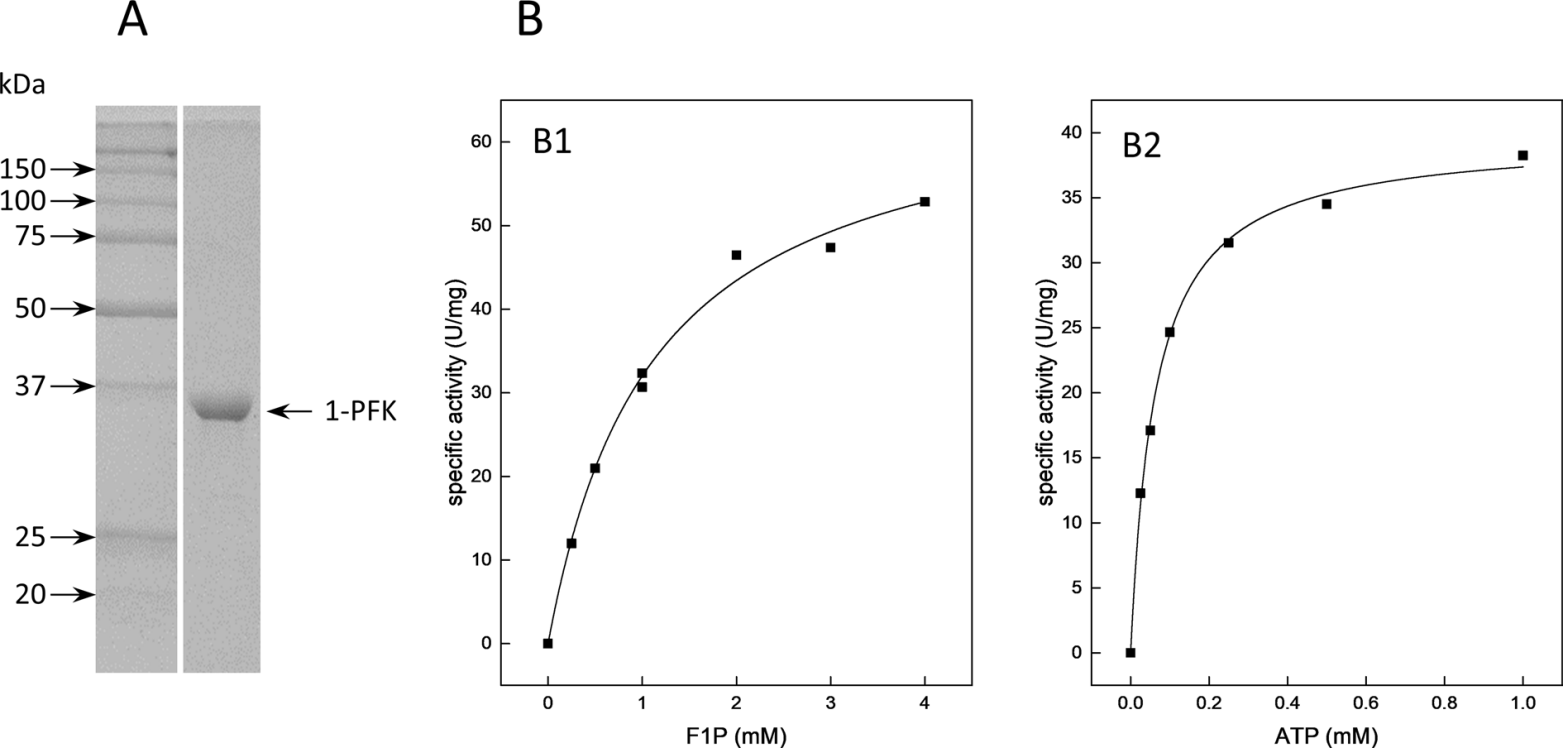
Purification and kinetic parameters of fructose-1-phosphate kinase (1-PFK) from *H. hispanica*. **A** SDS-PAGE of purified His-tagged 1-PFK*.* Arrows on the left side indicate molecular mass standards (kDa). **B** Rate dependence of 1-PFK on fructose-1-phosphate (F1P) (B1) and ATP (B2)

The Δ*fruK* mutant did not grow on mannitol and sorbitol, while growth on glucose was not affected (Fig. 2), indicating the involvement of 1-PFK in the degradation of both sugar alcohols by catalyzing the ATP-dependent phosphorylation of fructose-1-phosphate that is formed from fructose via ketohexokinase. The *fruK*-encoded 1-PFK was overexpressed in *H. volcanii* and purified by affinity and size exclusion chromatography (Fig. 9A). Recombinant 1-PFK catalyzed the ATP-dependent phosphorylation of fructose-1-phosphate (F1P) with a *V*_max_ of 67 ± 2.80 U/mg and *K*_M_ values of 1.1 ± 0.12 mM and 0.06 ± 0.004 mM for F1P and ATP, respectively (Fig. 9B). Phosphorylation of fructose-6-phosphate (F6P) was catalyzed with a *V*_max_ of 31 ± 5.8 U/mg and a *K*_M_ value of 34 ± 9.8 mM, i.e. at a 65-fold higher catalytic efficiency for F1P than for F6P, indicating a high specificity of the enzyme for F1P. The native molecular mass of 1-PFK, as determined by size exclusion chromatography, was 61 kDa characterizing the enzyme as a homodimer composed of 31.2 kDa subunits; a homodimeric oligomerization has also been described for the 1-PFK from *H. volcanii* (Pickl et al. 2012). The high sequence identity of 1-PFK from *H. hispanica* to the characterized 1-PFK from *H. volcanii* characterizes the enzyme as a member of the same enzyme family, the pfkB family of sugar kinases. Here, we were able to assign this kinase a novel in vivo function in the degradation of the sugar alcohols sorbitol and mannitol.

### Fructose-1,6-bisphosphate aldolase

The *fbaB1* gene (HAH_1079) encodes a putative FBA annotated as a member of Class I FBAs. A deletion mutant, Δ*fbaB1*, was not able to grow on sorbitol, mannitol, or fructose, while growth on glucose, as a control, was not affected (Fig. 2). This indicates the functional involvement of FBA in the degradation of mannitol and sorbitol. Also, the mutant did not grow on casamino acids (Figure S1) indicating an involvement in gluconeogenesis, as has been shown for FBA from *H. volcanii* (Pickl et al. 2012).

To demonstrate catalytic activity of FBA, a *H. volcanii* Δ*fba* mutant that is not able to grow on fructose (Pickl et al. 2012) was complemented *in trans* with *fbaB1* from *H. hispanica* (Figure S2). Indeed, growth of this complemented Δ*fba* mutant on fructose could be restored, indicating that the Class I FBA of *H. hispanica* shows catalytic activity in FBP cleavage and, furthermore, can replace the Class II FBA of *H. volcanii.*

**Fig. 10:**
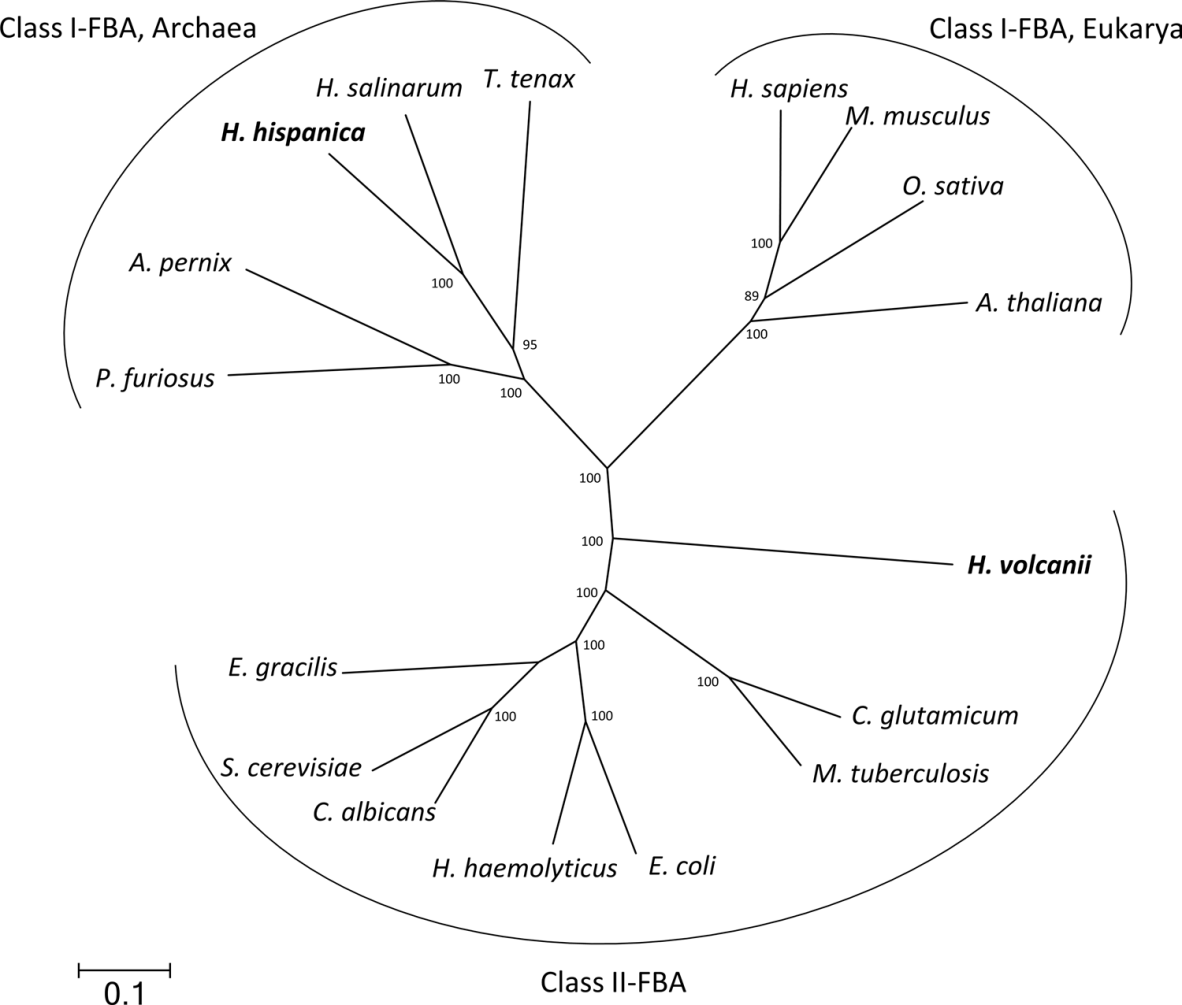
Phylogenetic relation of Class I fructose-1,6-bisphosphate aldolase (FBA) from *H. hispanica*, members of Class I FBAs from Archaea and from Eukarya, and of Class II FBAs from Archaea, Bacteria, and Eukarya. Class I FBAs from Archaea and from Eukarya and Class II FBAs form separate branches. FBA from *H. hispanica* clusters together with Class I FBAs from Archaea. UniProt accession: *Pyrococcus furiosus*, P58314; *Thermoproteus tenax*, P58315; *Haloarcula hispanica*, G0HX85; *Haloferax volcanii*, D4GYE0; *Aeropyrum pernix*, Q9YG90; *Halobacterium salinarum*, B0R3Y0; *Candida albicans*, Q9URB4; *Homo sapiens*, P04075; *Mus musculus*, Q91Y97; *Arabidopsis thaliana*, Q9SJU4; *Oryza sativa*, P17784; *Corynebacterium glutamicum*, P19537; *Escherichia coli*, P0AB71; *Euglena gracilis*, Q42729; *Haemophilus haemolyticus*, A0A0M3G3J9; *Mycobacterium tuberculosis*, P9WQA2; *Saccharomyces cerevisiae*, G2WHX2

Class I and Class II aldolases are classified by their reaction mechanism, catalyzing the FBP cleavage either via a Schiff base intermediate or the metal ion-dependent FBP cleavage without a Schiff base intermediate, respectively (Lorentzen et al. 2004). FBA from *H. volcanii* and from *Haloferax mediterranei* have been characterized as Class II members, while FBA from the closely related haloarchaeon *Haloarcula vallismortis* is described to be a Class I FBA (Krishnan and Altekar 1991; D’Souza and Altekar 1998; Pickl et al. 2012). A phylogenetic analysis of FBA from *H. hispanica* was performed with sequences of Class I FBAs from haloarchaea and archaeal homologs together with eukaryal Class I FBAs and Class II FBAs from Eukarya, Bacteria, and Archaea (Fig. 10). All three types of FBAs form distinct clusters, largely separated from each other. As expected, FBA of *H. hispanica* clusters together with archaeal Class I FBAs. In accordance, the *H. hispanica* FBA contains a lysine at position 177; a typical feature of archaeal type Class I FBAs, which is involved in the formation of a Schiff base intermediate for FBP cleavage (Lorentzen et al. 2004).

### Transcriptional regulation of mannitol and sorbitol degradation

**Fig. 11:**
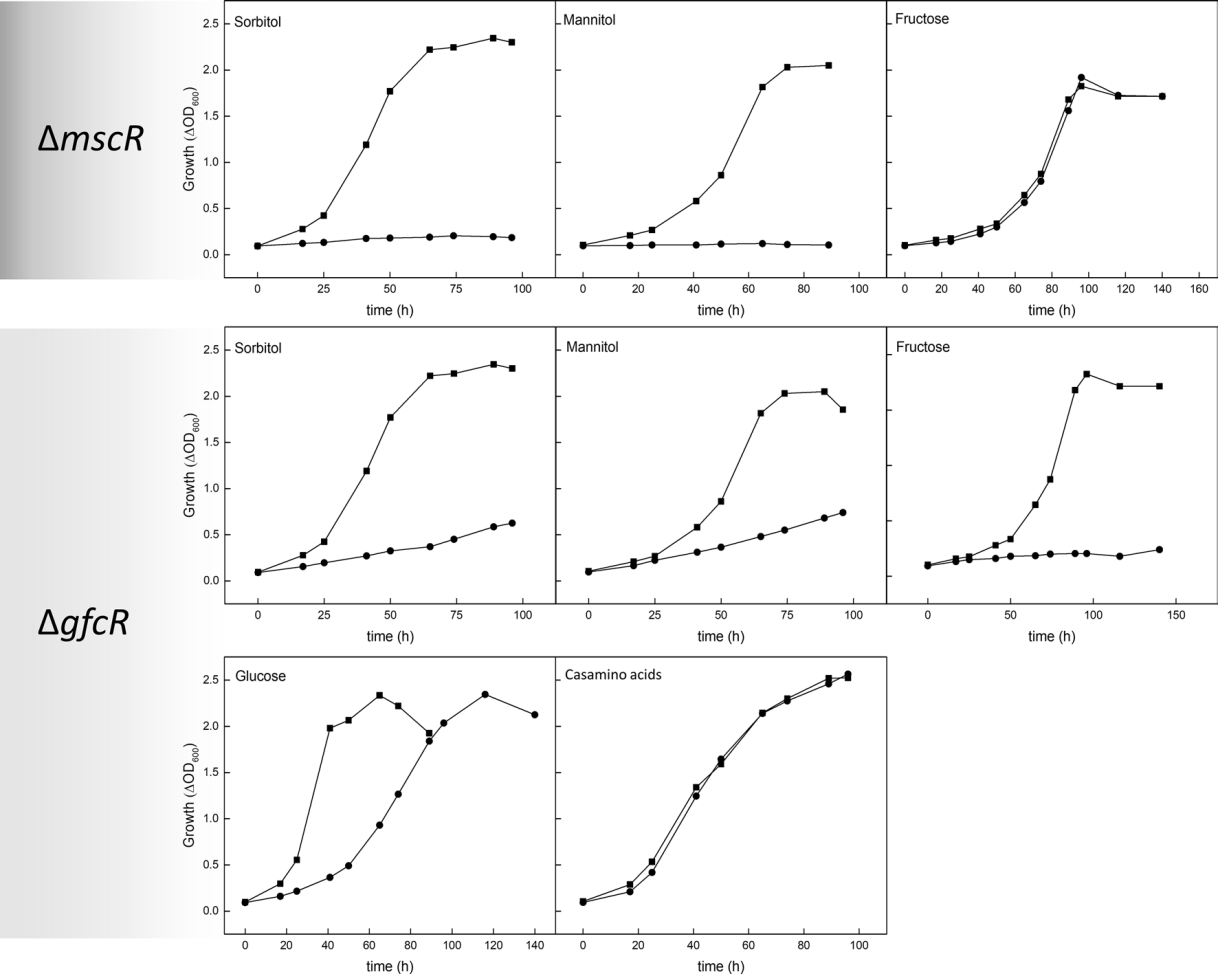
Growth analyses of deletion mutants of genes involved in the transcriptional regulation of enzymes of sorbitol and mannitol degradation in *H. hispanica*. Growth of deletion mutant strains (●) Δ*mscR* and of *ΔgfcR* was performed in comparison to the wild-type strain (■) on 10 mM each of sorbitol, mannitol, fructose, and glucose and on 1% casamino acids, respectively

In *H. hispanica,* the *msc* gene cluster, consisting of the *mscEFGK*, *mscS*, and *mscM* genes (Fig. 3), also comprises *mscR* (HAH_5148), which encodes a putative transcriptional regulator of the IclR family. A Δ*mscR* mutant was constructed and tested for growth on sorbitol, mannitol, and fructose, in comparison to glucose. The Δ*mscR* mutant was not able to grow on sorbitol and mannitol, while growth on glucose (not shown) and fructose was not affected (Fig. 11). These results suggest a function of MscR as a transcriptional activator of genes involved in the uptake of sorbitol and mannitol by the ABC transporter MscEFGK and in subsequent oxidation to fructose by SorDH and MtlDH. The finding that growth on fructose, which is an intermediate in sugar alcohol degradation in *H. hispanica*, was not impaired indicates that MscR is not an activator of genes involved in the degradation of fructose to pyruvate. MscR shows the typical two domain composition of IclR-like regulators described for Bacteria and haloarchaea, an N-terminal IclR-like helix-turn-helix domain for DNA binding (PROSITE accession: PS51077) and a C-terminal effector domain (PROSITE accession: PS51078) that potentially binds inducer molecules for activation of MscR (Figure S3). Regulators of the IclR family in Bacteria, e.g. *E. coli* and *Thermotoga maritima,* have been shown to regulate diverse metabolic processes including primary and secondary carbon metabolism, e.g. regulation of the glyoxylate pathway in *E.coli* and sporulation of *Streptomyces* (Molina-Henares et al. 2006). In recent years, various IclR-like regulators have been identified in haloarchaea functioning as activators of the catabolism of the sugars xylose, arabinose (XacR) and galactose (GacR), and of the deoxy sugar rhamnose (RhcR) (Johnsen et al. 2015; Reinhardt et al. 2019; Tästensen et al. 2020). Here, we present another IclR transcriptional regulator, MscR, that acts as an activator of the catabolism of the sugar alcohols, mannitol and sorbitol. Since the occurrence of IclR-encoding genes is restricted to bacteria and haloarchaea, it has previously been postulated that IclR-like regulators from haloarchaea originated from Bacteria by lateral gene transfer (Johnsen et al. 2015; Tästensen et al. 2020).

Recently, the novel transcriptional regulator GfcR, an activator of genes of glucose and fructose degradation pathways, has been identified in *H. volcanii* (Johnsen et al. 2023). A homolog of this protein was also identified in *H. hispanica*. This protein is encoded by *pyrE1* (HAH_1560), further designated as *gfcR* gene, and shows 63% sequence identity to GfcR from *H. volcanii*. A *gfcR* deletion mutant of *H. hispanica* did not grow on sorbitol, mannitol, and also not on fructose. Further, growth on glucose was impaired, while growth on casamino acids was not affected (Fig. 11). Since fructose is an intermediate in sorbitol and mannitol degradation, it is likely that GfcR in *H. hispanica* also acts as an activator of fructose degradation to triosephosphates by activating one or all genes of the fructose cluster, KHK, 1-PFK, and FBA, which has to be demonstrated. In *H. volcanii*, it has been shown that GfcR activates the genes of GAPDH I and pyruvate kinase; common genes of both glucose and fructose degradation (Johnsen et al. 2023). In conclusion, the degradation of sorbitol and mannitol in *H. hispanica* is likely to involve two different transcriptional activators: an IclR-like regulator activating genes of the *msc* cluster, i.e. those for mannitol/sorbitol uptake and oxidation to fructose, and a second, a GfcR-like regulator involved in the activation of genes of the fructose cluster encoding fructose degrading enzymes.

## Conclusion

The first report of growth of an archaeon on a sugar alcohol, mannitol, has been reported about 30 years ago for the haloarchaeon *Haloarcula vallismortis*. A degradation pathway has been postulated, but the enzymes involved were only partially characterized and their encoding genes have not been identified.

**Fig. 12:**
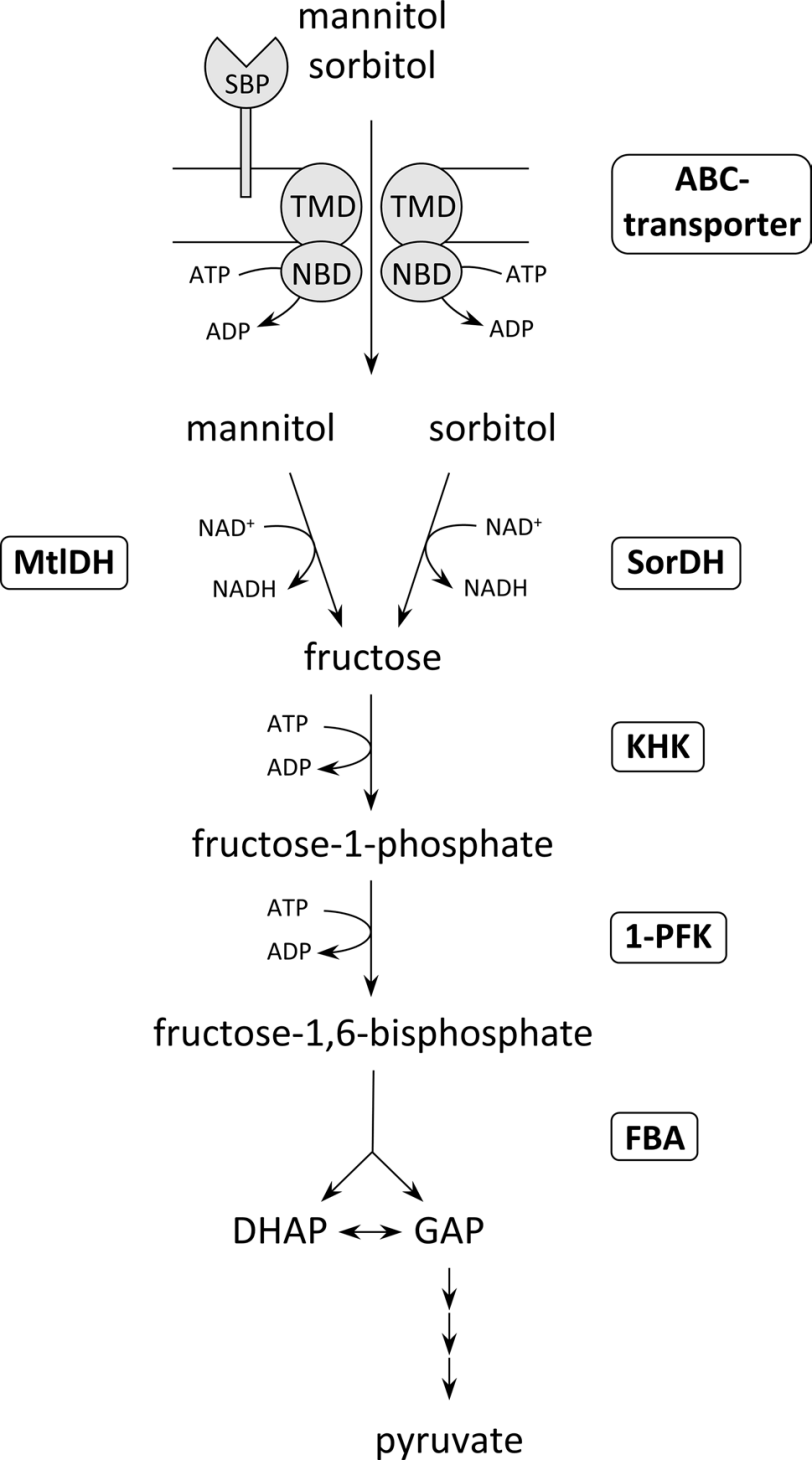
Proposed pathway of sorbitol and mannitol degradation in the haloarchaeon *Haloarcula hispanica*. Uptake of sugar alcohols is catalyzed by the same ABC transporter (SBP: substrate-binding protein; TMD: transmembrane domain; NBD: nucleotide-binding domain), yielding intracellular sorbitol and mannitol, which are oxidized to fructose via sorbitol dehydrogenase (SorDH) and mannitol dehydrogenase (MtlDH), respectively. Fructose is phosphorylated by ketohexokinase (KHK) to fructose-1-phosphate, which is phosphorylated to fructose-1,6-bisphosphate via fructose-1-phosphate kinase (1-PFK). FBP is cleaved to the triosephosphates dihydroxyacetone phosphate (DHAP) and glyceraldehyde-3-phosphate (GAP) that are further converted to pyruvate

Here, we present a comprehensive analysis of sugar alcohol degradation in the haloarchaeon *Haloarcula hispanica* (Fig. 12). The genes involved in uptake and degradation of mannitol and sorbitol were identified by growth experiments with deletion mutants, and key enzymes of the degradation pathway were characterized. It is shown that both alcohols are taken up by a promiscuous CUT1 ABC transporter and further oxidized to fructose by two distinct dehydrogenases, both members of the PDH family of the MDR superfamily. The subsequent degradation of fructose to FBA is catalyzed by ketohexokinase, 1-PFK and Class I type FBA. This is the first identification of an in vivo function of a KHK in prokaryotes, catalyzing the phosphorylation of fructose, originating from sorbitol and mannitol oxidation, to F1P. Further, we demonstrated that a Class I type FBA of *H. hispanica* can functionally replace a Class II type FBA from *H. volcanii.* Finally, we identified two transcriptional regulators that act as activators of sugar alcohol degradation; first, MscR is an IclR-like regulator activating genes of uptake and oxidation of sugar alcohols to fructose, and second, a GfcR-like regulator is involved in regulation of fructose degradation to pyruvate.

**Fig. 13:**
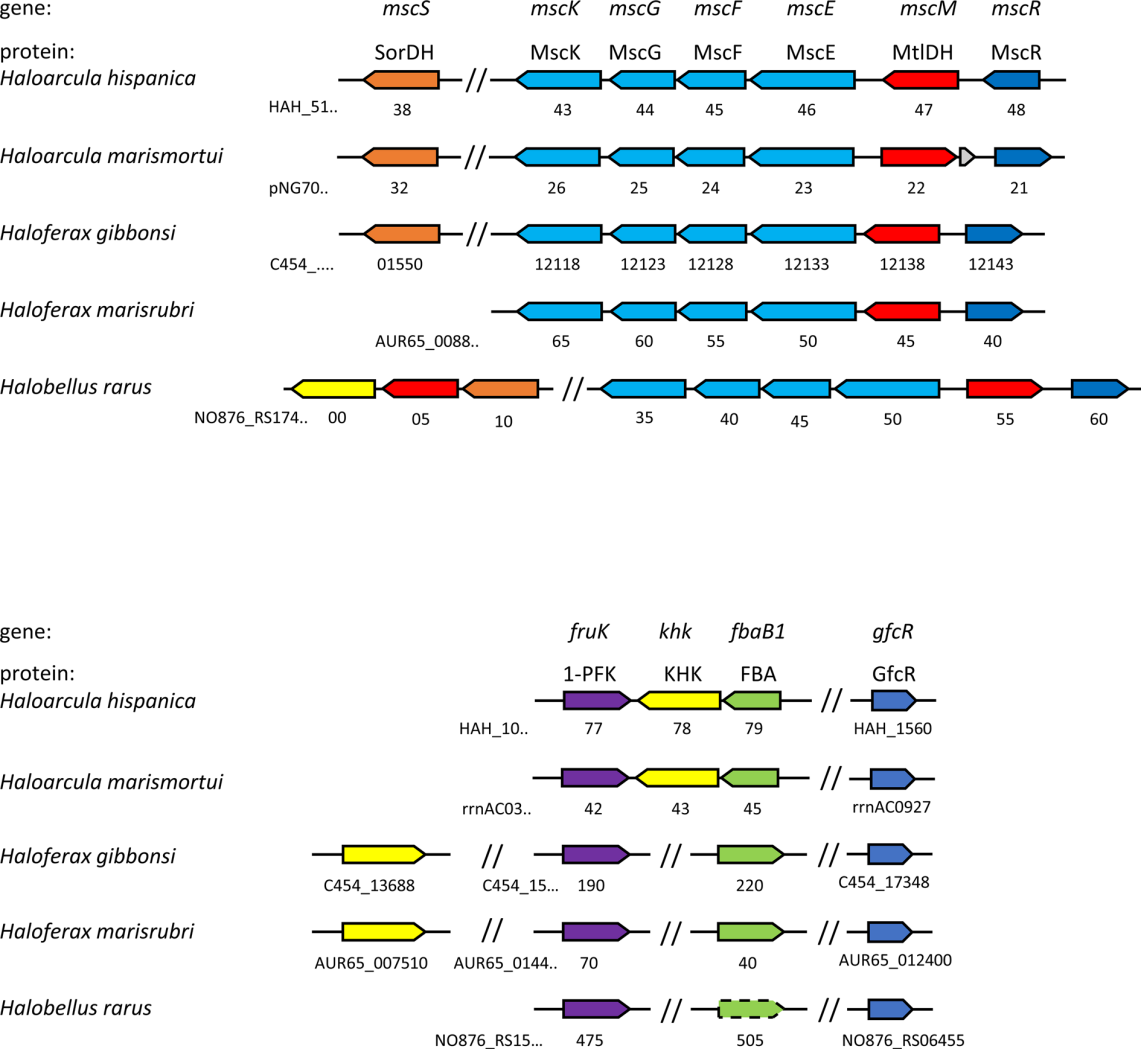
Genomic organization of gene clusters involved in uptake and degradation of sorbitol and mannitol in *Haloarcula hispanica* as compared to selected haloarchaea. Clusters and genes are shown for *H. hispanica*, *Haloarcula marismortui*, *Haloferax gibbonsi*, *Haloferax marisrubri*, and *Halobellus rarus*. Genes are indicated as colored arrows: sorbitol dehydrogenase (SorDH; *mscS*) in orange, ABC transporter (MscEFGK; *mscEFGK*) in light blue, mannitol dehydrogenase (MtlDH; *mscM*) in red, IclR-like transcriptional regulator (MscR, *mscR*) in dark blue, fructose-1-phosphate kinase (1-PFK, *fruK*) in purple, ketohexokinase (KHK, *khk*) in yellow, Class I fructose-1,6-bisphosphate aldolase (FBA, *fbaB1*) in green and GfcR-like transcriptional regulator (GfcR, *gfcR*) in dark blue. Class II aldolase of *Halobellus rarus* is indicated in green with dashed lines

Besides in *H. hispanica*, homologs of genes of the *msc* cluster and of the fructose cluster were also found in other haloarchaea, e.g. in *H. marismortui*, *Haloferax gibbonsi*, *Haloferax marisrubri*, and *Halobellus rarus* (Fig. 13). These findings indicate that the described pathway for sugar alcohol degradation in *H. hispanica* is also operative in other haloarchaea.

With the first characterization of a mannitol and sorbitol degradation pathway, we extend our knowledge of unusual sugar and sugar alcohol catabolism and its transcriptional regulation in the domain of Archaea.
